# An Improved Protocol for N-Glycosylation Analysis of Gel-Separated Sialylated Glycoproteins by MALDI-TOF/TOF

**DOI:** 10.1371/journal.pone.0015096

**Published:** 2010-11-29

**Authors:** Piliang Hao, Yan Ren, Yongming Xie

**Affiliations:** 1 Beijing institute of Genomics, Chinese Academy of Science, Beijing, China; 2 Applied Biosystems Inc. (China), Shanghai, China; University of South Florida College of Medicine, United States of America

## Abstract

Different glycoforms of some proteins have been identified as differential spots for certain diseases in 2-DE, indicating disease-related glycosylation changes. It is routine to determine the site-specific glycosylation of nonsialylated N-glycoproteins from a single gel spot, but some obstacles still exist in analyzing sialylated glycoproteins due to the lability and higher detection limit of acid glycans in MALDI-TOF/TOF analysis. Thus, we present an improved protocol here. Tryptic glycopeptides were separated and subjected to MALDI-TOF/TOF analysis, resulting in the identification of site-specific glycosylation of high-intensity glycopeptides. Sequential deglycosylation and desialylation were used to improve the identification of glycosylation sites and desialylated glycans. The site-specific glycosylation of large glycopeptides and low-intensity glycopeptides was deduced based on the masses of glycopeptides, deglycosylated peptides and desialylated glycans. By applying it to 2-DE separated human serum, the difference of N-glycosylation was successfully determined for α1-antitrypsin between different gel spots.

## Introduction

Of all known protein post-translational modifications (PTM), glycosylation is indeed the most common and complex one, which is predicted to occur on over 50% of all proteins and shows a great of diversities[1]. The glycans of glycoproteins are very important in many biological processes, such as embryonic development, cell division processes and protein regulations and interactions [2], [3]. The glycosylation status is crucial for protein functions, and it changes significantly during inflammation, sepsis and cancers [4]–[7]. It is important to monitor these changes and to find out how they happen. Glycoproteins must be separated from complex samples for detailed analysis. Despite that many technologies have been developed recently, two-dimensional electrophoresis (2-DE) remains one of the most efficient ways to conduct parallel comparison and separate complex samples [8], [9]. Thus, the development of glycosylation analysis method for gel-separated glycoproteins is imperative.

Presently, there are four types of known glycosylation, in which N-glycosylation and O-glycosylation are the most common ones. N-glycosylation occurs on asparagine residues in the consensus sequence Asn-Xxx-Ser/Thr (sometimes also Cys) *via* an *N*-acetylglucosamine residue, and Xxx can be any amino acid except proline. After digestion with PNGase F, the NH_2_ group in asparagine changes into OH group at the glycosylation site, and the mass of deglycosylated peptides increases 0.984 Dalton, which can be detected unambiguously with MALDI-TOF. In mammals, sialic acids are added by the capping reaction, and they are very important in cell-cell interactions. It is reported that the number of sialic acid moieties changes significantly during acute and chronic diseases, indicating that sialylated glycoproteins may be biomarker candidates [10], [11]. One sialylated glycoprotein is often separated into a string of spots with different p*I* values in 2-DE due to the different number of terminal sialic acids [12]–[14], and some of its glycoforms are identified as differential spots for certain diseases [4], [15]–[17]. Thus, it is important to elucidate the glycosylation information of the differential spots, which may be crucial in understanding the mechanism of related diseases and be identified as biomarkers.

Generally, glycoprotein characterization includes several steps, i.e. protein identification, determination of glycosylation sites and evaluation of site-specific microheterogeneities. Larsen et al. introduced a fast and sensitive method to characterize gel-separated nonsialylated N-glycoproteins [18]. However, it cannot be applied directly to sialylated glycoproteins because sialylated structures can lose a significant amount of sialic acids easily in the ion source or after the ion extraction from the ion source [2]. In recent years, many efforts have been made to analyze gel-separated sialylated glycoproteins. For example, von Witzendorff et al. identified the N-linked glycans of porcine zona pellucida glycoprotein ZPA with MALDI-TOF by desialylating them firstly, but it was impossible to determine the number of terminal sialic acids [19]. Permethylation or esterification of sialylated glycans could stabilize them and improve their detection with MALDI-TOF, but generally the amount of 2-DE gel-separated samples was insufficient for the treatments. A protocol using HILIC to extract glycopeptides was successfully employed in the analysis of gel-separated sialylated glycoproteins [20], but it failed to analyze large glycopeptides over 4000 Dalton and low-abundance glycopeptides.

Here we introduced an improved protocol to enhance the analysis of large glycopeptides over 4000 Dalton and low-abundance glycopeptides of which the site-specific glycosylation could not be determined with MS/MS. In this study, the composition of these glycopeptides was deduced based on the masses of glycopeptides, deglycosylated peptides and desialylated glycans. The sequential deglycosylation and desialylation greatly improve the detection of deglycosylated peptides and desialylated glycans. In addition, improved peptide desalting and neutral glycan identification methods were utilized in the protocol, which further enhanced its overall sensitivity. It was verified with 10 pmol gel-separated human transferrin and α1-antitrypsin spots from 2-DE separated human serum proteins.

## Materials and Methods

### Ethics Statement

The use of human sera for proteomics research was approved by the Institutional Review Board (IRB) of Beijing Institute of Genomics and informed consent was given by the participant in written.

### Materials

Human transferrin, α-cyano-4-hydroxycinnamic acid (CHCA), 2,5-dihydroxybenzoic acid (DHB), 2,4,6-trihydroxyacetophenone Monohydrate (THAP), diammonium citrate (DAC) and PNGase F were purchased from Sigma. Sialidase was purchased from Calbiochem. To pack microcolumns, HILIC material (ZIC™-HILIC, 200 Å, 10 µm, SeQuant AB, Sweden), Poros R2 material (20 µm, Applied Biosystems, USA) and graphitized carbon (obtained from dismantled Carbograph (Alltech, USA)) were packed in constricted GELoader tips (Eppendorf, Hamburg, Germany) as described [21]. Costar® Microcentrifuge tubes were purchased from Corning Incorporated. All other materials were purchased from Sigma unless otherwise described. The water was from a Milli-Q system (Millipore, USA). Human serum was taken from a healthy human.

### Gel Electrophoresis and In-gel Tryptic Digestion

For one-dimensional electrophoresis, 5 pmol and 10 pmol human transferrin was loaded, respectively. For 2-DE, 10 µL untreated human serum was loaded to each strip (7 cm, pH 4.7–5.9) for analyzing human α1-antitrypsin. The isoelectric focusing and 2-DE PAGE were conducted as described [4]. Proteins were fixed, stained and destained according to the cautions described by Kuster et al, and all the solutions should not contain over 7.5% acetic acid in order to preserve sialic acid-containing glycans [22]. Protein spots were then cut separately, and in-gel tryptic digestion was conducted as described except that trypsin was added at the ratio of 1∶30 [4].

### Enrichment of glycopeptides

Peptides and glycopeptides were extracted from gel particles under sonication for 20 minutes with 50% ACN containing 0.1% TFA twice [19]. The combined extracts were dried down in vacuo and redissolved in 80% ACN, 2% formic acid (FA). A 1-mL disposable syringe was used to force the liquid through self-packed microcolumns. After HILIC microcolumns were equilibrated by 80% ACN, 2% FA, the redissolved samples were loaded onto HILIC microcolumns slowly, washed with 40 µL equilibration buffer, and eluted with 2.5 µL 2% FA. About 0.6 µL glycopeptides were mixed with 0.6 µL DHB matrix on Bruker #25184 MALDI target and dried in air.

### Deglycosylation of glycopeptides

The rest of the HILIC enriched glycopeptides from the above step were dried in vacuo and redissolved in 20 µL 20 mM NH_4_HCO_3_, pH 8.0. To the solution, 1 µL PNGase F (5 U/100 µL) was added and incubated at 37°C overnight. One microliter of the digested samples was used to identify deglycosylated peptides on Bruker Anchorchip™ target.

### Desialylation of sialylated glycans

After the above-mentioned PNGase F digested samples passed through a Poros R2 microcolumn, the flow-through was treated with 0.05N HCl at 70°C for one hour to remove sialic acids [23]. Alternatively, sialidase could also be used to remove sialic acids. After the flow-through of Poros R2 microcolumn was dried and redissolved in 50 mM sodium acetate containing 1 mM CaCl_2_, pH 5.5, 5 µL sialidase (1 U/mL) was added. It was then incubated at 37°C overnight for complete removal of sialic acids.

### Purification of desialylated glycans

The glycan purification procedure followed previously described method [24]. After graphitized carbon microcolumns were equilibrated with water, the HCl treated or sialidase digested glycans were loaded into it slowly, washed with water, and eluted with 30% ACN, 0.1% TFA. The eluate was then dried in vacuo and redissoved in water.

### Matrix Preparation

The CHCA matrix was prepared by dissolving 4 mg of CHCA in 1 mL of 70% ACN/0.1% TFA. The DHB matrix was prepared by dissolving 10 mg of DHB in 1 mL of 50% ACN/0.1% TFA. The THAP matrix was prepared by dissolving 7.5 mg of THAP in 1 mL of ACN/20 mM ammonium citrate (1∶1 v/v).

### Sample preparation

For peptides, 0.5 µL samples were applied on AnchorChip™ target (Bruker Daltonics, Germany). After they were dried, about 0.1 µL CHCA matrix was added. The dried target was desalted by washing with 0.1% TFA twice and subjected to MS analysis. Glycopeptide samples were prepared as above mentioned. For glycans, 0.5 µL samples were applied to Bruker #25184 MALDI target, and then 0.5 µL of THAP was added. They were dried under vacuum (50×10^−3^ Torr) after mixed adequately with pipette tips. After vacuum drying, the sample appeared translucent. Upon absorption of moisture, the sample formed small crystals [25].

### MALDI-MS

MALDI-MS (/MS) data were obtained by an Ultraflex mass spectrometer (Bruker Daltonics, Germany). For peptide mass fingerprint (PMF) identification, the mass spectrometer was externally calibrated in order to reach the mass accuracy of 100 ppm. The database searches were performed against NCBInr database using Mascot software with fixed modification as carbamidomethyl (C) and variable modifications as oxidation (M). Desialylated glycans were analyzed in the same way with peptides. Glycopeptides were analyzed in the m/z range of 2000–7000 in the linear positive-ion mode. External calibration was performed using the average values of 5 tryptic peptides or glycopeptides of horseradish peroxidase, and the mass accuracy of <0.05% was obtained. Typically, spectra from 200 laser shots were added to obtain the final specta. The mass signals were manually annotated. MS/MS analysis in the TOF/TOF mode was conducted as described [4].

### Estimation of glycan composition and determination of glycopeptides

Estimation of N-linked glycan composition was accomplished by input of the peak masses into the GlycoMod Tool (http://www.expasy.org/tools/glycomod/). The masses of peptides and desialylated glycans were obtained in monoisotopic value in the reflectron mode of MALDI-TOF. However, because the masses of glycopeptides were obtained in average value in the linear mode of MALDI-TOF, the monoisotopic value of peptides and desialylated glycans must be turned into average values before calculation. Thus, we annotated their spectra manually with specific annotation method in order to get that average values. Finally, if the measured mass of a glycopeptide was equal to or 291*X (X = 1, 2, 3…) more than the predicted mass of a potential glycopeptide, i.e. different combinations of deglycosylated peptides and desialylated glycans, the potential glycopeptide should really exist, and the number of terminal sialic acids was X. The calculation is based on the fact that a glycopeptide is actually composed of three parts, i.e. the peptide part, the glycan part with sialic acids removed and the released sialic acids. The acceptable error was 500 ppm in the calculation.

## Results

### Principles of the Method

**Figure 1:**
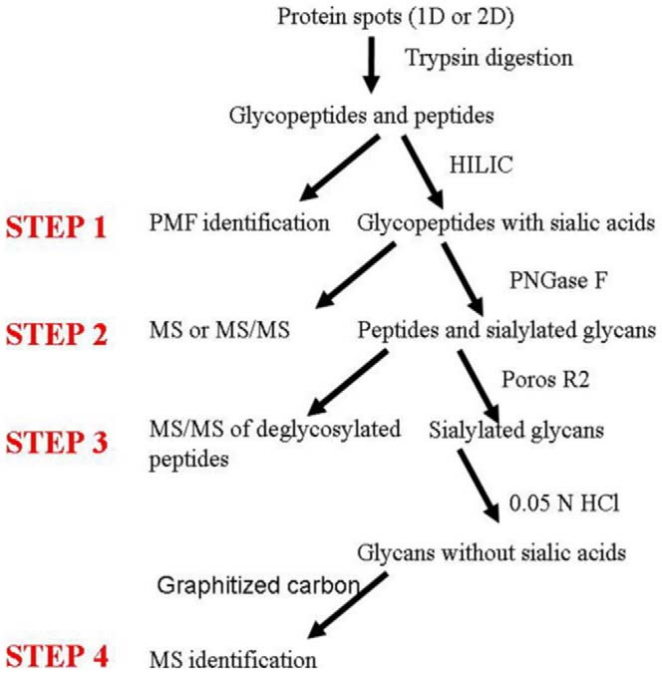
The strategy for N-glycosylation analysis of gel-separated sialylated glycoproteins.

**Figure 2:**
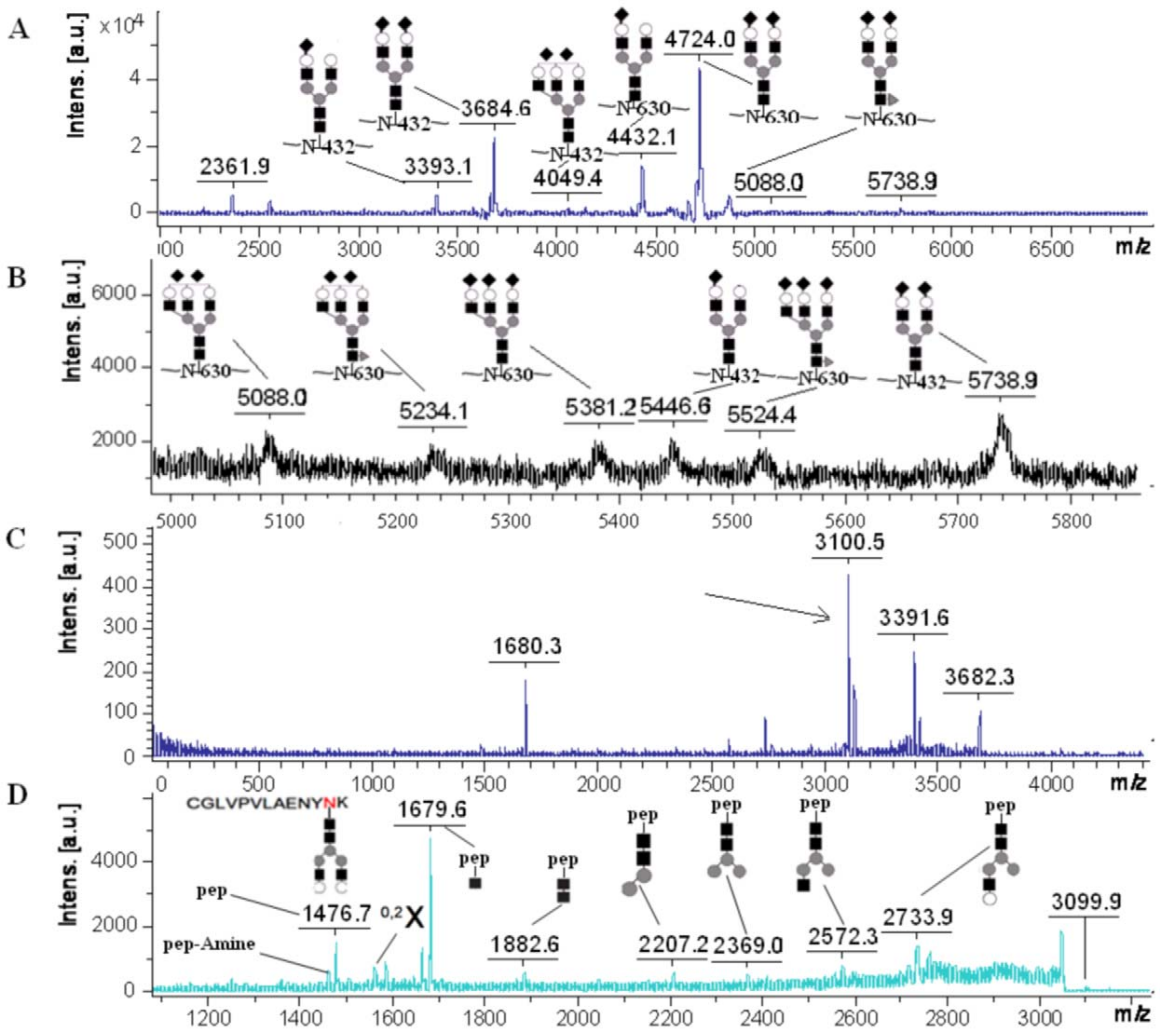
MS and MS/MS spectra of glycopeptides from 10 pmol gel-separated human transferrin. (A) Mass spectrum of the glycopeptides of human transferrin. (B) The enlarged view of Figure 2A, which shows low-abundance glycopeptides more clearly. (C) Mass spectrum of the parent ion of m/z 3684.6 obtained in TOF/TOF mode. The difference between 3682.3, 3391.6 and 3100.5 is about 291. (D) MS/MS spectrum from the precursor indicated by arrow in Figure 2C. The mass of [M_pep_+H]^+^ was 1476.7. Glycan structures were deduced based on the difference between adjacent signals and its biosynthesis process.

The strategy for analysis of gel-separated sialylated glycoproteins was illustrated in Figure 1. Briefly, after protein spots were digested by trypsin, some samples were used for PMF identification on Bruker Anchorchip™ target, which was proved to be efficient in peptide concentration [26]. We then predicted both potential N-glycosylation sites and the masses of potential deglycosylated peptides based on protein sequence. The MS signals of unmodified peptides tended to suppress that of glycopeptides, especially when glycopeptides contained sialylated glycans [27]. Thus, it was necessary to separate glycopeptides from other peptides by using HILIC materials, which was proved to be effective in glycopeptide enrichment [28]. Sialylated glycopeptides were generally analyzed in the linear mode of MALDI-TOF because of its easy loss of sialic acids in the reflectron mode[2]. However, as shown in Figure 2C and 2D, we found a new way to conduct MS/MS analysis on sialylated glycopeptides in the reflectron mode so that the number of terminal sialic acids, glycosylation sites and the linked glycans were obtained for high-intensity glycopeptides. The remaining glycopeptides were then deglycosylated with PNGase F. Some of the samples were used for MS and MS/MS analysis of deglycosylated peptides to confirm the glycosylation sites. After the remaining PNGase F digested samples passed through a Poros R2 microcolumn, we got the solution of sialylated glycans. Although Papac et al. reported that THAP was the best matrix in the analysis of sialylated glycans with MALDI-TOF [25], it was still impossible to identify sialylated glycans unambiguously from gel-separated proteomic level glycoproteins. Consequently, sialic acids were removed with HCl or sialidase digestion for enhancing the mass signals. Finally, desialylated glycans were purified with graphitized carbon microcolumns and analyzed with MALDI-TOF in the reflectron mode. After input of their masses into GlycoMod, we got their corresponding compositions. Because MS/MS analysis could not be effectively conducted on large glycopeptides or low-intensity glycopeptides, we had to predict their composition based on the masses of glycopeptides, deglycosylated peptides and desialylated glycans or deduce their composition based on their mass difference with the identified high-intensity glycopeptides. However, as shown in Figure 2C, we could also confirm the number of terminal sialic acids by collecting their parent ions in TOF/TOF mode. N-glycosylation analysis of gel-separated sialylated glycoproteins would be successfully accomplished with the protocol.

To demonstrate the efficiency of the strategy, human transferrin was chosen as the model protein because its glycosylation status has been analyzed in details in a multi-institutional study [29]. Compared to about 100 pmol samples used for glycopeptide characterization in that study, only 10 pmol samples were used in our protocol, and most low-abundance glycans around 1 percent as well as their glycosylation sites were successfully determined [29], indicating a significant increase of the detection sensitivity.

### PMF Identification

By conducting MS analysis on trypsin digested gel-separated samples, we got their MS spectra. Human transferrin was identified with the sequence coverage of 68%. Because N-glycosylation occurs in the consensus sequence Asn–Xxx–Ser/Thr (sometimes also Cys), we predicted potential glycosylation sites and the theoretical mass of the corresponding deglycosylated peptides from the sequence, i.e. 1477.744 (Asn-432), 3530.654 (Asn-432, with one missed cleavage), 700.328 (Asn-491) and 2516.117 (Asn-630). In the following, the prediction would be confirmed by MS or MS/MS analysis on deglycosylated peptides.

### MS and MS/MS analysis of glycopeptides

When glycopeptides enriched from gel-separated human transferrin were analyzed with MALDI-TOF, the mass spectra shown in Figure 2A–B were obtained. In a comparison to the recent published data [29], nearly all the glycopeptides of human transferrin were identified with much less samples except for the glycopeptide of m/z 4340.3, which owned a very low percentage in total. Most glycopeptides that owned about 1 percent in total were successfully identified with the improved protocol.

As shown in Figure 2C, when the precursor of m/z 3684.6 was collected with the LIFT method of Bruker Ultraflex, three major signals were obtained, i.e. m/z 3100.5, 3391.6 and 3682.3. The difference between the adjacent signals was about 291. Because sialic acids tended to lose when analyzed with MALDI-TOF in the reflectron mode, it was deduced that the glycopeptide had two terminal sialic acids. We then conducted MS/MS analysis on the glycopeptide losing sialic acids, i.e. m/z 3100.5. Generally, MALDI–TOF/TOF–MS of N-glycopeptides results in a set of cleavages at or near the innermost N-acetylglucosamine residue, with all the fragment ions retaining the peptide moiety so that four characteristic signals were generated, i.e. [M_pep_+H−17]^+^, [M_pep_+H]^+^, [M_pep_+H+83]^+^ and [M_pep_+H+203]^+^ [23], [30]. The characteristic peak pattern of delta 17-83-120Da is unique in the MS/MS spectrum of N-glycopeptides so that [M_pep_+H]^+^ can be determined, which leads to the identification of glycosylation sites. As shown in Figure 2D, because the deference between m/z 1459.6, 1476.7, 1559.7 and 1679.6 was about 17, 83 and 120 respectively, and m/z 1476.7 was equal to the predicted mass of certain unglycosylated peptide, i.e. CGLVPVLAENYNK (Asn-432), they were identified as the four characteristic signals in the MS/MS of N-glycopeptides. Thus, the glycosylation site of the glycopeptide was determined. The glycan structure of the glycopeptide was deduced based on the difference between adjacent mass signals in MS/MS spectrum and the biosynthesis process of glycans. We also conducted MS/MS analysis on m/z 4724.0 (data not shown). As with previous studies, its glycan composition could not be deduced from MS/MS because of insufficient dissociation, but the glycosylation site and number of terminal sialic acids were unambiguously identified. It is a significant improvement compared with previous analysis methods. The glycan composition of the glycopeptide was then determined based on its mass and that of deglycosylated peptides and desialylated glycans obtained later.

### MS/MS identification of deglycosylated peptides

**Figure 3:**
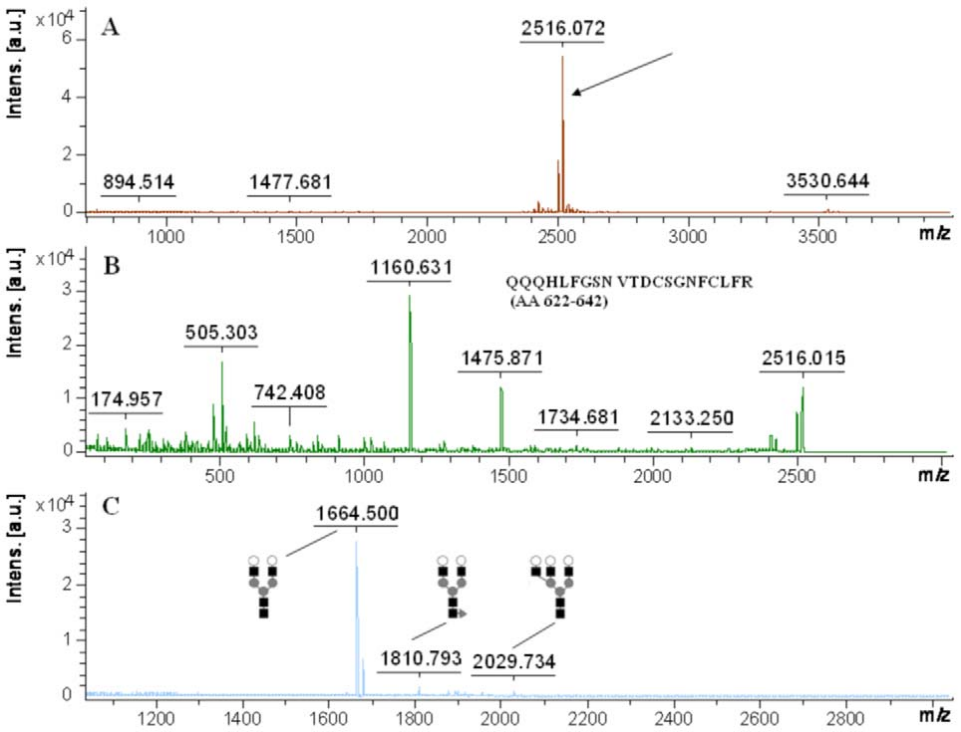
MS and MS/MS spectra of deglycosylated peptides and desialylated glycans of human transferrin. (A) Mass spectrum of deglycosylated peptides of human transferrin, m/z 1477.681, 2516.072 and 3530.644 were identified as deglycosylated peptides. (B) MS/MS spectrum of m/z 2516 indicated by arrow in Figure 3A. (C) Mass spectrum of desialylated glycans of human transferrin. The MS signals were [M+Na]^+^ ions in average values, and corresponding structures were also shown.

By comparing the masses of deglycosylated peptides in Figure 3A with their theoretical masses, m/z 1477.681, 2516.072 and 3530.644 were identified as deglycosylated peptides. MS/MS analysis was conducted on m/z 2516.072 to further confirm it, and the mass spectrum in Figure 3B was obtained. After searching the masses against NCBInr database using Mascot software, the peptide of QQQHLFGSDVTDCSGNFCLFR (Asn-630) was identified with a score of 87, and individual ions scores >34 indicate identity or extensive homology (p<0.05). These results further confirmed our prediction about glycosylation sites.

### MS analysis of desialylated glycans

We got the mass spectrum in Figure 3C while analyzing the graphitized carbon purified desialylated glycans using THAP as the matrix with MALDI-TOF in the reflectron mode. The composition of desialylated glycans was predicted based on the mass search in GlycoMod. For example, the composition of m/z 1664.500 was predicted to be (Hex)2 (HexNAc)2+ (Man)3(GlcNAc)2 with an error of −0.005 Da; that of m/z 1810.793 was (Hex)2 (HexNAc)2 (Deoxyhexose)1+ (Man)3(GlcNAc)2 with an error of 0.144 Da; that of m/z 2029.734 was (Hex)3 (HexNAc)3+ (Man)3(GlcNAc)2 with an error of −0.108 Da. The accuracy of the prediction is assured due to the small deviation from theoretical values. Their structures shown in Figure 3C were predicted based on the biosynthesis process of N-glycans in mammals and the comparison with known glycan structures.

### Assignment of glycopeptides

The calculation method mentioned in “Materials and Methods” was used to analyze glycopeptides. Since the masses of glycopeptides can only be obtained in average values in the linear mode of MALDI-TOF/TOF, the monoisotopic values of deglycosylated peptides obtained in the reflectron mode must be turned into average values before the calculation. As shown in Table 1, we firstly turned the monoisotopic value of deglycosylated peptides into average value, and then calculated M_PG_ (the masses of potential glycopeptides), M_PG_+291, M_PG_+291*2 and M_PG_+291*3 respectively. After comparing these values with the measured masses of glycopeptides from human transferrin, most glycopeptides were identified except for m/z 5234.1 and 5525.9. Base on the difference between m/z 5234.1 and 5088.7 and that between m/z 5234.1 and 5525.9, they were also identified (See also Figure 2B). The number of terminal sialic acids was confirmed by MS/MS analysis of low-intensity glycopeptides. Now, all the glycopeptides were identified for gel-separated human transferrin.

**Table 1:** The mass of potential glycopeptides of human transferrin calculated from that of deglycosylated peptides and desialylated glycans.

[M_pep_+H]^+^_mono_	[M_pep_+H]^+^_avg_	[M_glycan_+Na]^+^_avg_	[M_PG_+H]^+^_avg_	[M_PG_+H]^+^_ avg_ +291	[M_PG_+H]^+^_ avg_ +291*2	[M_PG_+H]^+^_ avg_ +291*3
1477.681(Asn-432)	1478.700	1664.5001810.7932029.734	3101.2003247.4933466.434	**3392.200**3538.4933757.434	**3683.200**3829.493**4048.434**	3974.2004120.4934339.434
2516.072(Asn-630)	2517.773	1664.5001810.7932029.734	**4140.273**4286.5664505.507	**4431.273** **4577.566**4796.507	**4722.273** **4868.566** **5087.507**	5013.2735159.566**5378.507**
3530.644(Asn-432)	3532.958	1664.5001810.7932029.734	5155.4585301.7515520.692	5446.4585592.7515811.692	**5737.458** **5883.751**6102.692	6028.4586174.7516393.692

M_PG_: Mass of potential glycopeptides.

Note: The measured masses of the glycopeptides of human transferrin are 3393.0, 3684.6, 4049.4, 4142.9, 4432.1, 4576.6, 4724.0, 4871.2, 5088.7, 5234.1, 5381.8, 5525.9, 5738.8 and 5884.1. The values in bold are identified glycopeptides.

### N-glycosylation analysis of 2-DE gel-separated human α1-antitrypsin

**Figure 4:**
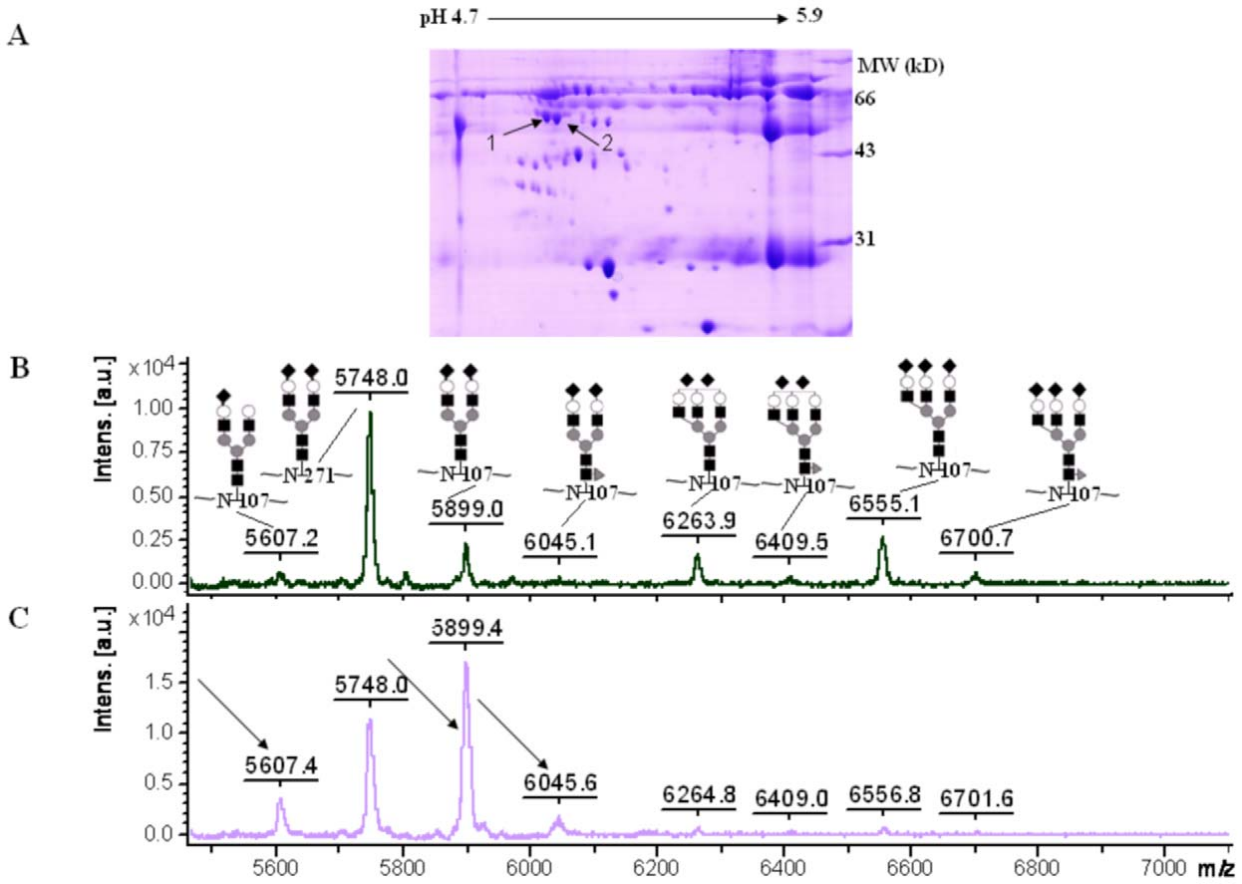
2-DE image of 10 µL human serum and mass spectra of glycopeptides of 2-DE separated α1-antitrypsin. (A) 2-DE image of 10 µL human serum. Two largest spots of α1-antitrypsin were chosen for glycosylation analysis and named as A1PI-1 and A1PI-2. (B)–(C): Mass spectra of the glycopeptides of A1PI-1 and A1PI-2 in the mass range of 5500-7000 Dalton. Data were shown in average value. Compared with A1PI-1, the relative abundance of diantennary N-glycans indicated by arrows in A1PI-2 increased significantly, while the triantennary N-glycans decreased significantly.

In order to prove that our protocol was also applicable to 2-DE separated samples, we applied it to human α1-antitrypsin from 10 µL normal human serum separated by 2-DE (Figure 4A). The two largest spots of α1-antitrypsin were chosen for analysis, and they were named as A1PI-1 and A1PI-2 from low p*I* to high p*I*. N-glycans on all three glycosylation sites of human α1-antitrypsin were determined successfully for both spots using our improved protocol. The mass spectra of the glycopeptides of A1PI-1 and A1PI-2 were listed between 5500 and 7000 Dalton in Figure 4B and 4C because they had differences only in the range. Compared with A1PI-1, the relative abundance of diantennary N-glycans indicated by arrows in A1PI-2 in figure 4C increased significantly, while that of the triantennary N-glycans decreased significantly. The relative abundance of each glycan on Asn107 was listed in Table 2. The glycosylation analysis of 2-DE separated human α1-antitrypsin indicated that there were more triantennary glycans with two or three terminal sialic acids and less diantennary glycans in 2-DE spots with lower p*I* for the same protein, which was consistent with previous research work [31].

**Table 2:** Relative abundance (%) of the glycans identified at Asn107 of human α1-antitrypsin a.

	I	II	III	IV	V	VI	VII
CarbohydrateStructure on Asn107	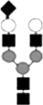	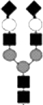	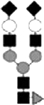	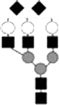	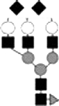	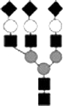	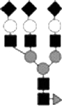
A1PI-1	7.4%	25.8%	4.8%	19.3%	4.8%	30.2%	7.8%
A1PI-2	14.9%	69.9%	7.2%	2.9%	1.3%	2.8%	1.1%

a Approximate abundance was estimated using MS signal intensities from a single analysis.

## Discussion

Both N-glycosylation sites and site-specific microheterogeneities were determined unambiguously from 10 pmol gel-separated human transferrin band using the improved protocol. We also tried 5 pmol samples as the lower limit. Although glycopeptides could also be identified, it was insufficient for the subsequent deglycosylation and desialylation. It was a little difficult to get 10 pmol samples from 2-DE gel spots, but sufficient samples could be obtained for glycosylation analysis by applying different amount of samples on gels for protein identification and glycosylation analysis, respectively [32]. By applying the presented protocol to human lactoferrin, a widely studied glycoprotein, 6 new low-abundance glycans were discovered (unpublished data), which validated the sensitivity of the protocol. The desialylation treatment improves the detection of N-glycans greatly.

In the past, many researchers used to desalt peptides by Poros R2 or graphite powder before MALDI-TOF analysis. It is a simple and convenient method. However, in this study, Bruker Anchorchip™ target was utilized in desalting peptides because its workflow was more simple and time-saving, which is consistent with the great number of proteomic protein identification. Furthermore, it is better in preserving small hydrophilic peptides that are generally lost during the desalting with other methods. Thus, higher protein sequence coverage was obtained in PMF identification [26]. In the enrichment of glycopeptides, HILIC is more efficient than the generally used agarose bound lectins in the nano-scale analysis because of its smaller particle size (10 µm vs 200–300 µm), which is consistent with the extremely low amount of samples from 2-DE spots. Moreover, HILIC enriches glycopeptides because of their hydrophilic property but not specific glycan structures. Thus, it is the material of choice for overall glycopeptide enrichment [30]. Recently, magnetic beads functionalized with di-boronic acid, ConA and WGA were developed by Bruker Daltonics Inc., but they could not be used in the nano-scale enrichment of glycopeptides [33].

Papac et al. reported that 50 fmol sialylated glycans were detected with signal-to-noise ratio of better than 5∶1 with MALDI-TOF when THAP was used as the matrix[25], but it was still impossible to identify sialylated glycans unambiguously from a single gel-separated sialylated glycoprotein spot. The possible explanation is that when the amount of proteins is in the low picomole level, the loss of them is much more significant during the preparation, such as attachment on GEloader tips and pipette tips. Thus, we chose to desialylate acid glycans in order to improve their detection. The desialyation with HCl was quick and efficient. However, some contaminant signals less than 1500 Dalton appeared occasionally. The possible reason is that the polypropylene tubes may degrade at the high temperature of 70°C. Thus, a glass bottle should be a better choice. Alternatively, if sialidase was used for desialylation, the occurrence of contaminant signals could be avoided, but the incubation period was much longer.

Generally, many researchers used DHB or Super-DHB as the matrix in the analysis of neutral glycans [12], [25], [34]. However, based on our results, THAP/DAC provides a better result in the MS analysis of neutral glycans, which achieves better signal-to-noise ratio and signal intensity with same amount of glycans. And it also provides better shot-to-shot and sample-to-sample reproducibility. However, it has not been widely employed yet because it is rather difficult to get small crystals while using THAP as the matrix. The mixture of glycans and THAP must be dried immediately in high vaccum after mixed completely. Otherwise, large crystals will form, and no mass signals of glycans can be obtained.

One disadvantage of our protocol is that it provides no direct information about monosaccharide type and glycan linkage type. Generally, it needs to conduct permethylation, NMR research or sequential glycosidase digestion on glycans in order to get the detailed information, but gel-separated samples are surely insufficient for the treatments. In our protocol, glycan composition was determined accurately. Considering the biosynthesis process of glycans and known glycan structures in public databases, we could also get their structures. The site-specific glycosylation of large glycopeptides and low-abundance glycopeptides was mainly deduced from MS data but not from MS/MS due to the limitation of current mass spectrometers. In future, it is expected that MS/MS can be successfully done on these glycopeptides with MALDI-TOF/TOF with the quick development of mass spectrometers.

Our protocol has extended the previous protocol to the analysis of large glycopeptides over 4000 Dalton and low-abundance glycopeptides. The sequential deglycosylation and desialylation improve the identification of glycosylation sites and glycans greatly. It is sensitive enough to analyze gel-separated sialylated glycoproteins, especially different glycoforms of the same glycoprotein separated by 2-DE. It can also be used in the glycosylation analysis of purified or recombinant glycoproteins due to its excellent sensitivity. It is reported that many potential biomarkers for cancers and inflammation are glycoproteins with different glycosylation status [10], [11], [35]. The elucidation of glycosylation status of glycoproteins will be helpful to understand the mechanism of certain diseases and identify potential biomarkers.
